# Congenital Partial Colonic Agenesis in Dogs and Cats: Clinical, Biological, Diagnostic Imaging, Endoscopic and Histopathologic Characterization, a Retrospective Study

**DOI:** 10.3390/vetsci10090577

**Published:** 2023-09-18

**Authors:** Paul Remmel, Lucile Gros, Jérémy Mortier, Valérie Freiche

**Affiliations:** 1 Unité de Médecine Interne, Centre Hospitalier Universitaire Vétérinaire d’Alfort (CHUVA), École Nationale Vétérinaire d’Alfort, 94700 Maisons-Alfort, France; valerie.freiche@vet-alfort.fr; 2 Service d’imagerie Médicale, Département d’Élevage et Pathologie des Équidés et Carnivores (DEPEC), Centre Hospitalier Universitaire Vétérinaire d’Alfort (CHUVA), École Nationale Vétérinaire d’Alfort, 94700 Maisons-Alfort, France

**Keywords:** congenital, colonic agenesis, stenosis, canine, feline

## Abstract

**Simple Summary:**

Partial colonic agenesis is a rare congenital disorder of the large intestine. However, no study has reported its presentation in dogs and cats yet. The aim of this article is to retrospectively describe the clinical, biological, diagnostic imaging and endoscopic findings of 23 cases of partial colonic agenesis. Although agenesis is a congenital disease, the age of presentation was variable and long asymptomatic periods were common. Abdominal ultrasound was useful and identified the agenesis in 14/17 cats whereas clinical and biological data remained unspecific. Endoscopy allowed precise measurements and diagnosis but also the identification of concurrent lesions. The most frequent associated lesion was colonic stenosis, which might represent a complication of the disease. This study highlighted that partial colonic agenesis could be considered as a possible cause of chronic diarrhea in dogs and cats. Its early diagnosis might prevent further complications such as colonic stenosis.

**Abstract:**

Congenital diseases of the large intestine of dogs and cats have scarcely been reported and mostly include fistula, atresia or colonic duplication. Cases of partial colonic agenesis have rarely been described. The purpose of this study was to report a cohort of dogs and cats diagnosed with partial colonic agenesis. The colon was measured during colonoscopy or contrast-radiography and compared to the average length described in the literature. Six dogs and 17 cats were retrospectively included. Depending on the case, partial colonic agenesis could represent an incidental finding or the likeliest cause of clinical signs. Diarrhea was reported in most cases, and no specific clinical or biological abnormality was observed. Median age of presentation was variable and long asymptomatic periods were common. Abdominal ultrasound was useful and identified a short colon in 14/17 cats but only in one dog. Endoscopy was useful to confirm the diagnosis and to identify associated lesions and complications. Among others, colonic stenosis was reported in 8/9 cases that had lifelong clinical signs and the shortest colon length. This anatomical abnormality could promote chronic inflammation that might generate fibrosis and ultimately stenosis.

## 1. Introduction

The colon is the terminal part of the digestive tract; it begins at the ileocolic sphincter and ends at the rectum. It is composed of the ascending, transverse and descending parts, demarcated by connecting flexures [1]. Its main roles are absorption and secretion of water and electrolytes, storage of feces, immune surveillance, and it is the site of microbial fermentation [2]. Colonic diseases are relatively frequent disorders in dogs and cats. In young animals, the main etiologies are infectious (parasites), alimentary (food intolerance, fiber responsive colopathy) or breed-related (granulomatous colitis), according to Veterinary Medicine. Congenital colopathies are rare and mostly include fistula [3,4], atresia [5] or colon duplication [6]. The normal length of the colon varies between 60 to 75 cm in dogs (ranges 25–90) [7,8] and is approximately 30 cm in healthy cats (27–37 cm) [9]. It may depend on the size of the animal and is usually considered to contribute to 20–25% of the total intestinal length [1,2,9]. Very few data about congenital partial colonic agenesis exist in the literature. Previous studies have described isolated case reports of short colon in a foal [10] and in few dogs and cats [11,12].

The objective of this study was to retrospectively describe a population of dogs and cats diagnosed with congenital partial colonic agenesis. 

## 2. Materials and Methods

Partial colonic agenesis was defined as a colon length with at least a 15% reduction compared to the lowest values described in the literature for healthy dogs and cats [7,8,9].

In cats, a threshold of 22 cm was retained (15% reduction of 27 cm). In dogs, there is an extreme variability in body weight and size that affects the colon length with values ranging from 30 cm in dogs weighing 5 kg to 99 cm in dogs weighing 60 kg [13,14]. As such, a threshold of 25 cm (15% reduction of 30 cm) was used to define partial colonic agenesis for small dogs (below 15 kg). For medium size dogs (between 15 and 30 kg), a 15% reduction of the average length described was used (60 cm, [7,8]). A threshold of 50 cm was retained (15% reduction of 60 cm). We did not record dogs heavier than 30 kg in this study.

Medical records of all cats and dogs diagnosed with partial colonic agenesis between January 2016 and May 2022 at the Veterinary Teaching Hospital of Alfort, Paris, France, were retrospectively searched from the database. 

**Figure 1:**
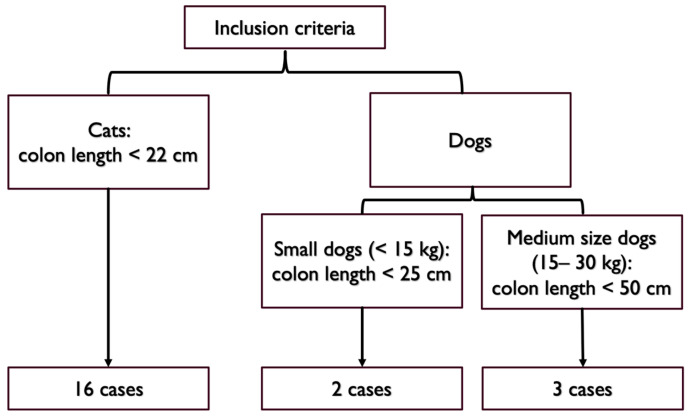
Colon length thresholds used for inclusion criteria.

Inclusion criteria included the measurement of a short colon defined by our thresholds using contrast radiography, ultrasonography, endoscopy, or a combination of these modalities (Figure 1). Cases with a colon length above threshold were excluded. Two cases with an extremely short colon on abdominal ultrasound did not have endoscopy performed yet were also included (one cat and one dog).

Information was collected regarding signalment, clinical signs, age at presentation, clinical signs duration, diagnostic tests, treatments, and outcome. Hematologic data (scil Vet abc, IDEXX ProCyte Dx), serum biochemical analysis (Scil VetScan, IDEXX catalyst), electrolytes, B9 and B12 vitamin profile and stool analysis from patients at presentation or at the referring veterinarian were assessed. Abdominal ultrasonography was performed with an Affinity 70, Phillips Medical System, using both microconvex and linear probes. Normal ranges for ultrasonographic measures were defined according to the current literature [15,16,17]; among others, colonic wall was considered thickened above 1.5 mm for dogs and cats and lymph nodes were considered enlarged over 5 mm for dogs and 4 mm for cats. Subjective assessment based on lymph node echogenicity and shape was also taken into consideration for equivocal cases. Abdominal ultrasonography and abdominal radiographs were reviewed by the same board-certified radiologist (L.G.). Endoscopic evaluation was performed by the same operator (V.F.) with an Olympus Evis Exera III Gif-h190 gastrointestinal videoscope and according to the WSAVA endoscopic recommendations [18]. At least six colonic biopsies were performed. Histological specimen were also evaluated according to the WSAVA criteria [18]. Medical treatment and outcomes were compared when available. 

To differentiate cases with lifelong clinical signs from cases with an onset during adulthood, we distinguished cases with clinical signs lasting since the adoption (referred thereafter as Group A) and cases with emerging clinical signs (referred thereafter as Group B). As animals might have been adopted during adulthood, we recorded the age of adoption. For animals in Group B, we calculated the ratio between the duration of clinical signs (in months) and the age (in months). Cases for which no primary colonic lesion but the partial colonic agenesis was detected were described together as Subgroup C (see below).

Data were expressed by standard descriptive statistics and presented as mean or median and range (minimum-maximum). The Student’s *t*-test was used to compare age between Groups A and B. Categorial variables were compared among groups using the chi-squared test. Each of which initially included variables that were significant at *p* < 0.05 in simple logistic regression. Statistical analyses were performed using available statistical software (BiostatGV, http://biostatgv.sentiweb.fr/, accessed on 6 September 2023).

## 3. Results

### 3.1. Epidemiological and Clinical Data

A total of 23 cases, 17 cats and six dogs, were included in the study. Among the cats, there were seven females and 10 males and among the dogs, there were four females and two males. Males and females comprised 12 and 11 of the 23 cases, respectively. Subject to the statistical representativeness of our population, no correlation was identified between sex, and the occurrence of a partial colonic agenesis (*p*-value: 0.88; chi-squared test, considering a 50% prevalence of males and females in the healthy population). Two French bulldogs and two boxers were present among the six dogs. The other breeds were one border collie and one whippet. Domestic short hair (DSH) was the most common breed in cats (5/17). Most cats were purebred and included Bengals (3/17) and one of each of the following breeds: Siberian, Savanah, exotic shorthair, Persian, Birman, Scottish, Burmese, Abyssinian and Chartreux. Epidemiological data, living conditions, deworming and vaccination status are reported in Table 1.

**Figure 2:**
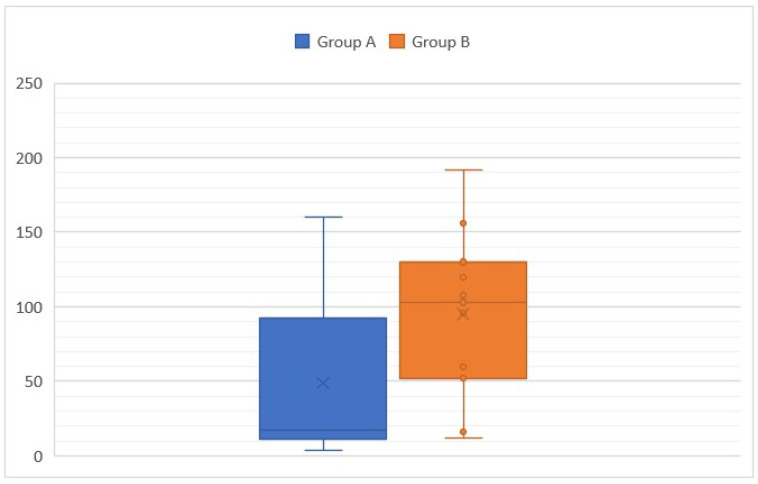
Age at presentation according to the group (A: animals with clinical signs lasting since the adoption and B: animals with a progressive or acute onset of clinical signs during adulthood).

The median age was 81 months/6.7 years in cats and 56 months/4.7 years in dogs. The age distribution was bimodal: one group with clinical signs lasting since the adoption (Group A) and one group with a progressive or acute onset during adulthood (Group B). In Group A, the mean age at presentation was 48.8 months/4 years with a median of 17 months/1.4 years and a range of [4–160] months. This group included 11/17 cats and 1/6 dogs. The median age of adoption in this group was 3 months with a range of [2–96] months. Only three cases were adopted during adulthood. In Group B, the mean age was 95 months/7.9 years with a median of 103 months (range, [12–192] months). Mean and median duration of clinical signs in this group were 14.5 months and 12 months (range, [4–48] months), respectively. This group included 5/6 dogs and 6/17 cats. Age of adoption was not consistently recorded for this group. In Group B, the ratio between the duration of clinical signs and the age was consistently below 0.5 and indicated silent periods before presentation. Age was not significantly different between Groups A and B (Figure 2) (Student’s *t* test). 

The most common clinical signs were diarrhea (21 of 23 cases/91.3%) and weight loss/failure to grow (11 of 23 cases/47.8%). Table 2 summarizes the clinical signs. Table 3 and Table 4 summarize the clinical signs in Groups A and B. All clinical signs were present in both groups except for dysorexia or polyphagia, which were only found in Group B. Mucoid stools, urgency to defecate and dyschezia were significantly more common in Group A (prevalence in Table 3 and Table 4, *p*-value < 0.001, 0.03 and <0.001, respectively). 

Upon physical examination, a low body condition score was the most common abnormality. It was slightly low (BCS of 3/9) in seven of 23 cases (30%) and markedly low in two cases (BCS of 2/9, 8.7%). It was normal in 12 of 23 cases (52.2%, BCS 4–5/9) and increased in two (8.8%, BCS 6–7/9). Thickened intestinal loops were noticed on abdominal palpation in three of 23 cases and one case had a painful abdominal palpation. One case presented with lethargy, hyperthermia and dehydration. In most cases, the physical examination was unremarkable (17 of 23 cases, 73.9%). 

### 3.2. Diagnostic Tests

#### 3.2.1. Hematochemical Analysis

Blood cell counts were performed in 21 of 23 cases and revealed slight nonregenerative normocytic normochromic anemia (PCV 26%, reference range [29–48]%) in three cats; neutrophilic leucocytosis in two cases (19,500 and 22,190 cells/mm^3^); mild eosinophilia in five cases (median: 1300; range [1250–1690] cells/mm^3^), monocytosis in four cases (median: 1 485; range [1040–2030] cells/mm^3^), basophilia in one case (260 cells/mm^3^) and lymphocytosis in one case (10,380 cells/mm^3^).

Biochemistry tests were performed in 20 of 23 cases and revealed slight hyperuremia in one case (0.88 g/L; reference range [0.4–0.8] g/L); increased ALT in one dog (700 U/L; reference range [15–123] U/L); increased ALP in one dog (410 U/L; reference range [22–187] U/L); increased AST in one cat (62 U/L; reference range [0–45] U/L); decreased albumin in three cases (18–25 g/L; reference range [26–35] g/L); hypoglobulinemia in one case (24 g/L) and hyperglobulinemia in two cases (51 and 67 g/L; reference range [25–45] g/L).

Electrolytes were measured in 16 cases. It revealed hyponatremia (135 mmol/L; reference range [150–165] mmol/L) in one of 16 cases and hypokalaemia (3.2–3.4 mmol/L; reference range [3.6–5.5] mmol/L) in two of 16 cases.

Trypsin-like-immunoreactivity (TLI) was measured in eight cases and was normal in all but one cat.

Cobalaminemia was measured in 18 cases. It was decreased in three cases and increased in eight cases. Folic acids were measured in seven cases, increased in four and decreased in one case.

Fecal analysis was performed in 15 cases (three dogs and 12 cats). It comprised 13 fecal flotations on three-day pooled samples and two PCR tests for Giardia in two cats. Results were negative in all cases where it was tested. One cat had a positive tritrichomonas PCR out of nine cases tested. Prophylactic treatment with fenbendazole was given in four out of the eight cases that had not a stool analysis performed.

In Group A, hypercobalaminemia was present in five of 10 cases; hyperfolatemia was present in two of five cases; hypocobalaminemia in one of 10 cases and hypofolatemia in one of five cases. Biochemical abnormalities were present in three out of 10 cases with one case of mild hypoalbuminemia, one case of mild hyperuremia and one case of moderate hyperglobulinemia. The five cases with eosinophilia and the case with basophilia were all in Group A. 

In Group B, hypercobalaminemia was present in three of eight cases; hyperfolatemia was present in two of two cases and hypocobalaminemia in one of eight cases. Biochemical abnormalities were mostly present in Group B: one increase in ALT, one increase in ALP, one increase in AST, one mild hyperglobulinemia, two decreases in albumin and one hypoglobulinemia.

In Subgroup C, hypercobalaminemia was present in four of eight cases; hyperfolatemia was present in one of three cases; hypocobalaminemia in one of eight cases and hypofolatemia in one of three cases. Biochemistry was unremarkable for this group. The five cases with eosinophilia and the case with basophilia were in Group C. 

#### 3.2.2. Abdominal Ultrasound

Abdominal ultrasound was performed in 22 cases. Peritoneal effusion was present in seven of 22 cases and was associated with hypoalbuminemia in one case. A partial colonic agenesis was suspected from abdominal ultrasound in 15 cases (14 cats and one dog).

Colonic wall thickness was increased in 17 of 22 cases. In Group A, it was increased in nine of 11 cases. In Group B, it was increased in eight of 11 cases. It was focal in four cases and diffuse in 13 cases. Table 5 summarizes the subjective severity of colonic wall thickening and its distribution depending on groups. Loss of colonic wall layering was observed in four cases.

The ileocolic junction was visualized in 20/22 cases and did not show any abnormality. One case (canine) had a thickened cecal wall.

Ileocolic or colic lymphadenomegaly was seen in 14 cases (seven in Group A and seven in Group B).

Other ultrasound abnormalities included gall bladder wall thickening (three cases), hypoechogenic liver (three cases), gastric wall thickening (two cases) and small intestinal wall thickening (eight cases). These other abdominal ultrasound abnormalities were more common in Group B (12/16 in Group B). 

#### 3.2.3. Abdominal Radiographs

**Figure 3:**
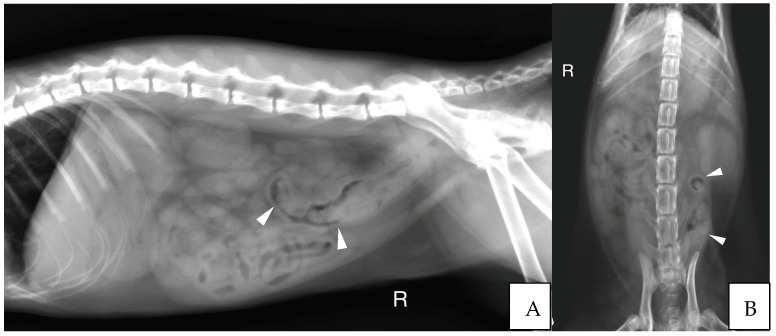
(**A**) Right lateral radiograph and (**B**) Ventro-dorsal radiograph of the abdomen of case 11, a six months old Scottish cat. The descending colon is shorter than expected and abruptly ends in the mid left abdomen (white arrow heads). No other parts of the colon are identified. R: Right.

**Figure 4:**
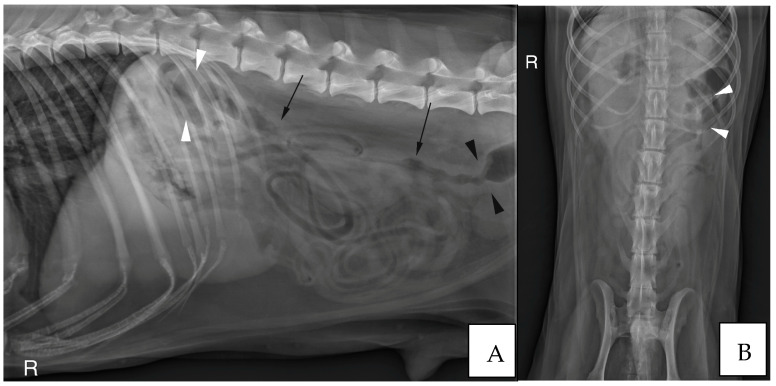
(**A**) Right lateral radiograph and (**B**) Ventro-dorsal radiograph of the abdomen of case 18, an eight years-old Collie dog. The cecum is malpositioned in the left cranial and dorsal abdomen (white arrow heads), in continuity with the descending colon on the lateral view (black arrows). A focal narrowing of the aboral part of the descending colon is observed before it enters the pelvis (black arrow heads). R: Right.

**Figure 5:**
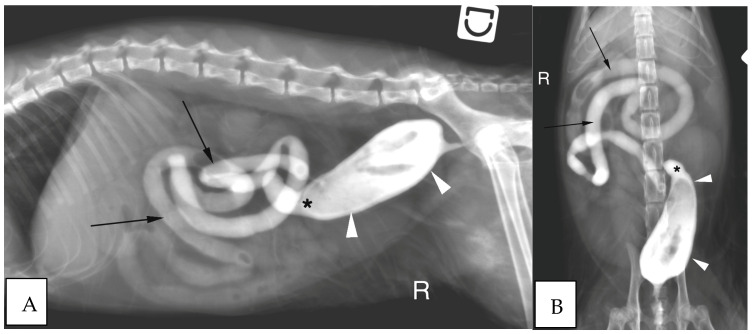
(**A**) Right lateral radiograph and (**B**) Ventro-dorsal radiograph of the abdomen of the same cat as on image 2, during positive retrograde colonography. The descending colon is shorter than expected (white arrow heads), the ileocolic junction is identified as a narrowing of the colonic lumen orally (asterisk). Contrast medium is also visible within the small intestine (black arrows). The cecum is not identified. R: Right.

Abdominal radiographs were acquired in five cases (four cats and one dog). In two cats, the descending colon showed an abrupt ending with a total length approximately half as long as expected. No ascending nor transverse colon could be identified in these two cases (Figure 3). In the dog, the cecum appeared mispositioned in the left cranial abdominal quadrant and seemed associated with a focal area of colonic lumen narrowing 22 cm orally to the anus (Figure 4). Positive contrast colonographies were performed in three cases (three cats), and allowed the diagnosis of partial colonic agenesis in all of them (Figure 5).

#### 3.2.4. Endoscopic Findings

Colonic length was available in 19 cases. In cats, colonic length ranged from eight to 20 cm with a median of 18 cm. Four cats had a marked colon shortening with lengths ranging from eight to 12 cm whereas 10 had only mild partial shortening with values ranging from 18 to 20 cm. In dogs of 10 kg or less (two cases), colonic length ranged from 22 to 23 cm. In dogs over 15 kg (three cases), it ranged from 40 to 45 cm.

**Figure 6:**
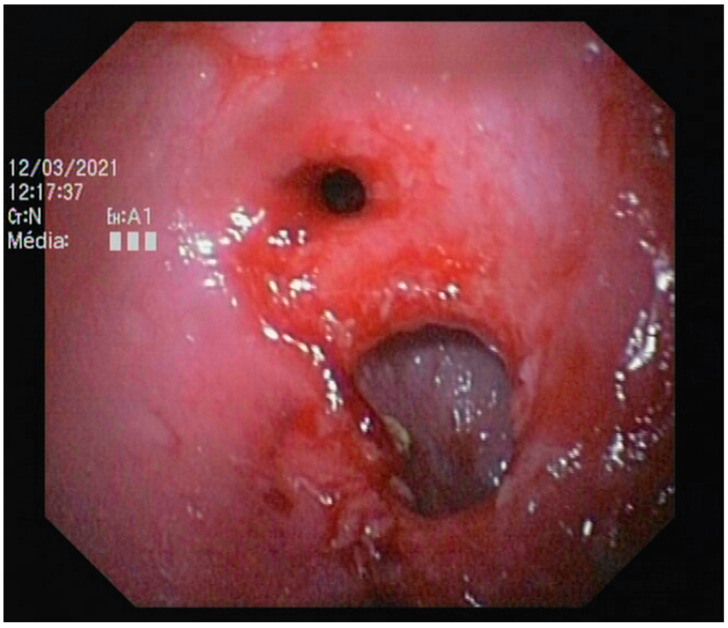
Abnormal ileocolic junction in a cat (Case 10).

**Figure 7:**
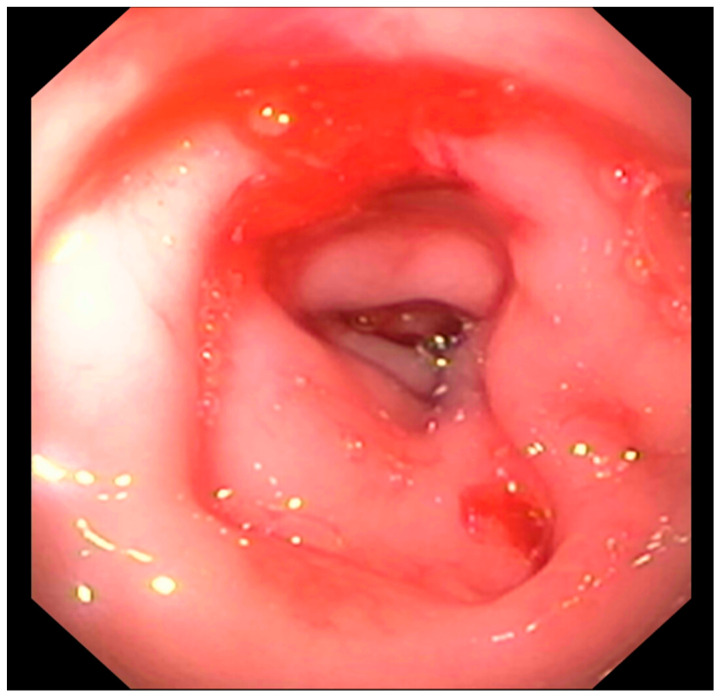
Focal colonic stenosis in a cat (Case 15).

Endoscopic abnormalities are presented in Table 6 and included: a gaping, atone and opened ileocolic junction in eight cases (four in Group A and four in Group B, Figure 6), absence of cecum in one case, colonic stenosis of variable degrees in 12 cases (two in Group B and 10 in Group A, Figure 7), erosive or marked macroscopic signs of colopathy in five cases (four in Group B and one in Group A), non-specific, slight-to-moderate signs of colopathy in eight cases (three in Group B and five in Group A) and an unremarkable colon in two cases (one each in Groups A and B). 

#### 3.2.5. Histology

Histology was available in 20 cases (Table 7). It revealed a lymphoplasmacytic infiltrate in 19 cases, that was mild-to-moderate in 16 cases (six in Group A and 10 in Group B) and marked in three cases (two in Group A and one in Group B). A neutrophilic infiltrate was present in nine cases (five in Group A and four in Group B); erosion or ulcers were found in six cases (three in Group A and three in Group B) and fibrosis was seen in six cases (four in Group A and two in Group B). Two cases showed a significant eosinophilic infiltrate (Group B). 

#### 3.2.6. Diagnosis

A concurrent diagnosis was established in all the cases in Group B and comprised one low grade intestinal T-cell lymphoma in a cat (LGITL), one protein-losing-enteropathy in a dog (PLE) and four granulomatous colitis. In the five remaining cases, a non-specific, mild-to-moderate lymphoplasmacytic infiltrate was present and was considered secondary to an inflammatory bowel disease (IBD) based on an exclusion diet. 

One dog in Group A had a diagnosis of granulomatous colitis, one cat had trichomoniasis and one cat had a granulomatous infiltrate. The latter showed marked polyclonal hyperglobulinemia, developed neurological signs and displayed high anti-coronavirus antibodies titres that led to the suspicion of feline infectious peritonitis. 

**Figure 8:**
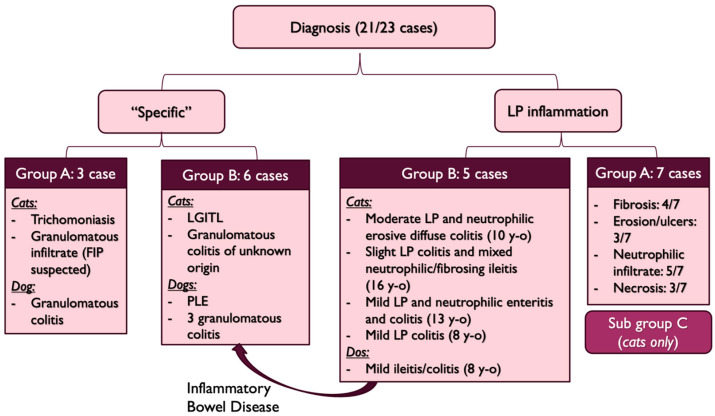
Diagnosis repartition (FIP: Feline Infectious Peritonitis, LGITL: Low Grade Intestinal T-cell Lymphoma, PLE: Protein-Losing-Enteropathy, y-o: years-old, LP: lymphoplasmacytic, y-o: years old).

Seven other cases had a lymphoplasmacytic infiltrate of variable severity associated with fibrosis in 4/7 cases, neutrophilic infiltrate in 5/7 cases, mucosal erosion or ulcers in 3/7 cases and necrosis in 3/7 cases. In these seven cases, no other diagnosis was found and since the clinical signs lasted since adoption, they were categorized as “Subgroup C” (Figure 8). All were cats and all but one developed a stenosis. 

The last two cases were cats that did not have histological analysis performed due to owner’s decline and could not be assigned to a specific diagnosis group. However, they had lifelong clinical signs, marked shortening of the colon with stenosis and they both improved with lactulose and hyperdigestible diet or interventional treatment (colonic stenting) without relapse. Hence, the clinical signs were attributed to the stenosis and the partial colonic agenesis, and these cats were also included in the Subgroup C.

Cats of this subgroup had a significantly shorter colon than the other cats (*p* = 0.04, Student’s *t*-test, median length 13 cm vs. 18 cm, range [8–20] cm vs. [15–20] cm). 

#### 3.2.7. Management and Outcome

Treatment depended on the presence or absence of a stenosis and on the nature of the mucosal infiltrate: neutrophilic or ulcerative/erosive cases led to antibiotics whereas lymphoplasmacytic infiltrates led to diet changes and/or corticosteroids. Specific diseases (LGITL, PLE, tritrichomonosis, granulomatous colitis) were treated with the respective treatment (chlorambucil and prednisolone, prednisolone, ronidazole, fluoroquinolones, respectively). Antibiotic sensitivity tests were performed in all but one case before fluoroquinolone prescription; otherwise, antibiotic treatment was mostly empirical for amoxicillin/clavulanic acid, metronidazole or tylosin. The data are presented in Table 8.

Diet changes for hyperdigestible or hydrolysed diets and symptomatic treatments were usually first intended and failed to resolve clinical signs.

Stenosis was managed by partial colectomy (two cases), bougienage (two cases) or endoluminal stenting (one case). Neutrophilic or ulcerative/erosive cases were managed by antibiotics (amoxicillin/clavulanic acid in two cases, metronidazole in six cases, marbofloxacin in two cases, enrofloxacin in two cases, and tylosin in one case). Antibiotics were also used in a case of cholecystitis and suspected acquired dysbiosis.

Prednisolone was prescribed in cases of lymphoplasmacytic infiltrate and ranged from 0.5 mg/kg/d to 2 mg/kg/day. Budesonide was used in one case. Other treatment included chlorambucil (one case), ciclosporin (one case) and were all in Group B, depending on additional diagnosis.

Six cases were lost to follow-up. Three cases were included during the redaction of the manuscript and had no follow-up data. Two cases died of unrelated causes and one died because of persistent marked diarrhea and hypovolemic shock. One case relapsed after six months of normal stools and was diagnosed with colonic adenocarcinoma (ADK). In the remaining cases, the four cases with suspected granulomatous colitis on histology improved with fluoroquinolone antibiotherapy. Two cases with a diagnosis of IBD and PLE improved with immunosuppressive treatment and one case with LGITL improved with cytotoxic treatment. 

In Subgroup C (seven cases), four were lost to follow-up, two did not have follow-up at the time of redaction yet, and one died of post-operative complications after a colectomy for colonic stenosis.

Two cases showed marked stenosis and had no histological analysis performed. One improved with an endoluminal stent and prednisolone and the other one with lactulose, metronidazole and a hyperdigestible diet.

**Figure 9:**
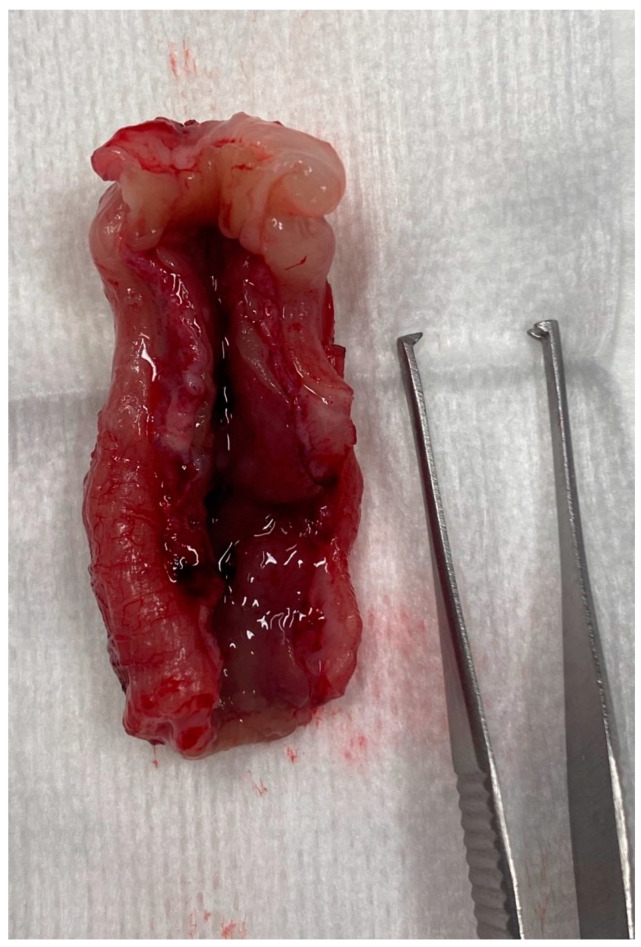
Colectomy in a cat with partial colonic agenesis: the total length was estimated to 8 cm.

One case underwent necropsy which confirmed partial colonic agenesis (Figure 9). No other malformation was found on the digestive tract. 

## 4. Discussion

To the authors’ knowledge, this is the first study describing congenital partial colonic agenesis in a cohort of dogs and cats. In this study, there was no sex predisposition and too few cases to detect breed overrepresentation. However, cats included in the Group C were mostly purebreed cats.

This study included 17 cats and six dogs. Eleven cats had clinical signs since adoption (Group A) and six had silent period before presentation (Group B). One dog belonged to Group A and five to Group B. It is unknown why the malformation was more often diagnosed in cats. A possible explanation might be that there is a much greater variation of size in dogs and thus, of colon length considered normal; which might have led to the inclusion of only severe cases. Moreover, all dogs had a concurrent diagnosis. It is thus possible that this malformation might have been overlooked and considered as an incidental finding that did not participate to the clinical signs.

Ordered by frequency, clinical signs included diarrhea, hematochezia, weight loss or failure to thrive, vomiting, mucoid stools, dyschezia and dysorexia. No clinical signs nor specific associations of clinical signs were found to be specific in the diagnosis of a partial colonic agenesis, even if more pets in Group A had signs of colitis.

Likewise, CBC, biochemistry and electrolytes were non-specific. Mild anemia, monocytosis and mild neutrophilia were occasionally present and likely secondary to chronic inflammation. Five cases had mild eosinophilia that was not explained by parasitism or by an eosinophilic infiltrate in the colon. Biochemical abnormalities were seldom. Apart from one dog that had panhypoproteinemia due to PLE, all biochemical abnormalities remained mild and were considered non-specific (chronic inflammation, dehydration, suspected reactive hepatitis) in the context of gastro-intestinal disease. Cobalamin and folic acid abnormalities were variable and could not be predictive of a partial colonic agenesis or of any group. No association was found between hypocobalaminemia and ileocolic valve malformation. One cat had low TLI consistent with exocrine pancreatic insufficiency (EPI). In this case, EPI did not seem to explain the dyschezia [19], therefore motivating colonoscopy that led to the diagnosis of partial colonic agenesis.

Abdominal ultrasonography seems to be a useful tool in the diagnosis of partial colonic agenesis, leading to the diagnosis in 14 of 17 cats. However, diagnosis of partial colonic agenesis was more challenging in dogs; abdominal ultrasonography identified it in only one case, suggesting a lack of sensitivity of this modality for diagnosing this disease in dogs. This apparent discrepancy could be explained by the challenge of localizing the ileocecal junction in dogs compared to cats, which indirectly reflects the colon size. The ileocolic junction and the caecum are typically located to the right of the midline, along with the duodenum [1]. This localization might be modified in a case of partial colonic agenesis and might raise the suspicion of a shorten colon. On canine abdominal ultrasound however, the junction can be challenging to identify because the cecum is most commonly gas-filled. On the contrary, in cats, it can be more easily recognized [15]. Thus, an abnormal position of the ileocolic junction in association with a subjective short colon length might be more easily recognized in feline cases and increases the suspicion of partial colonic agenesis. Unfortunately, due to the retrospective nature of the study, the imaging criteria that led the radiologist to suspect a short colon were not available. Thickening of the colonic wall and local adenomegaly were seen in 17 and 14 cases of 22, respectively, but are unspecific findings in cases of colic diarrhea. Few cases had other abdominal ultrasound abnormalities outside of the colon or associated lymph nodes and were more common in Group B (12/16 in Group B). They may thus, be secondary to a concomitant disease.

Abdominal radiography was performed in few cases and raised the suspicion of partial colonic agenesis in only half of them. This difference might be due to the difficulty in identifying the oral part of the colon on abdominal radiograph because its filling with gas or stool is variable. Positive colonography has not been investigated extensively in this study. However, as the ileocolic junction was clearly identified in the two cases with colonography, it might be a useful and easily accessible tool to diagnose partial colonic agenesis in small animals.

Colonoscopy allowed the diagnosis in most cases. Partial colonic agenesis was associated with other anatomical malformations in eight cases and included absence of the cecum or abnormal ileocolic junction (Figure 6). Focal stenosis was also identified in 12 cases. Marked macroscopic signs of active colonic disease were identified in five cases and corresponded to a diagnosis of granulomatous inflammation or IBD. In most cases however, the colic mucosa was macroscopically normal or only slightly modified, especially in Group A. 

Two different subtypes of presentation were identified in this study: one with clinical signs that lasted since a young age or adoption, and one with clinical signs that developed during adulthood. In cases where clinical signs developed in adult individuals, a concomitant affection was present in all cases and included IBD, PLE, LGITL, and granulomatous colitis. It is likely that these diseases were responsible for the clinical signs and that the partial colonic agenesis was an incidental finding. However, it might also have been a predisposing factor to the development of these diseases. Indeed, in these 11 cases, an infectious origin was suspected or confirmed in four cases (granulomatous infiltrate). A partial colonic agenesis might then have increased to sensitivity of the colon to infectious agent.

In cases where clinical signs started young or at adoption, an infectious agent was found in three cases and included one trichomonosis and two granulomatous infiltrates. In the nine remaining cases (Subgroup C), seven were submitted to a histologic analysis that showed variable degrees of lymphoplasmacytic inflammation. Eight out of these nine cases developed a colic stenosis. This complication has previously been described in one case report of short colon [12]. In this case, the cat had chronic and progressive signs of diarrhea and developed dyschezia. Colonoscopy identified stenosis with mild-to-moderate lymphoplasmacytic infiltration and fibrosis, as well as absence of an ileocolic papilla. It is then possible that, in these individuals, partial colonic agenesis might have led to a persistent colic inflammation which predisposed them to secondary fibrosis and stenosis. In dogs and cats, rectal strictures can indeed be secondary to diarrhea and the associated colonic inflammation [20], and in humans, stenosis is a well-known complication of Crohn’s disease [21]. The pathogenesis involves repetitive and unphysiological healing of injured tissue after chronic exposure to inflammatory factors. These factors lead to smooth muscle hyperplasia and activate fibroblasts which produce excessive amount of growth factor and fibrous matrix proteins. Expansion of smooth muscle layers and collagenous fibres deposition progressively lead to gut lumen narrowing and ultimately stenosis [22,23,24]. 

In the Subgroup C, the length of the colon was significantly shorter than in the other cats of the cohort. However, overlap exists, and it remains unclear why some individuals might develop severe clinical signs, whereas some remained free of clinical signs for several years. In cats and dogs who underwent small intestinal resection, secondary morphological modifications allow an increase of the villous mucosal surface area following the length reduction [25,26]. This also seems to be the case when the resection affects the colon with an adaptative response with villi elongation in the small intestine after sub-total colectomy in dogs [27]. In all our cases with clinical signs since adoption (with the exclusion of infectious cases), the histological analysis revealed only mild-to-moderate lymphoplasmacytic infiltration. It is then possible that a histological compensatory adaptation was lacking in this group that could explain the early presentation. Comparison with asymptomatic cases of partial colonic agenesis would be required to answer this question.

In Group A, the median age of adoption was young (3 months) with three cats adopted at the ages of one, two and eight years old. The presence or absence of clinical signs before adoption for these three cases remains unknown. However, they all belonged to the Subgroup C, where no concurrent diagnosis was made and clinical signs attributed to the short colon length. As such, we assumed that their clinical signs might have occurred since a young age. This assumption is a limitation and further work is needed to confirm that such cases do not present a silent period.

Thus, we believe that cats with lifelong clinical signs of colitis and unremarkable or unspecific hematochemical analysis might be evocative of a partial colonic agenesis, and that these cats are at higher risk of developing colonic stenosis or of being subjected to infectious processes. This malformation probably also exists as an incidental finding in both dogs and cats with milder forms and silent periods.

The outcomes were highly variable in our cases. Because many of them were lost to follow-up, it is difficult to draw conclusions on the potential benefits of the treatments initiated (Table 8). However, in some cases, management of the clinical signs seemed to be more difficult than in animals exhibiting a colopathy without partial colonic agenesis.

Partial colonic agenesis might be part of a wider syndrome related to a short bowel. Indeed, congenital short bowel is described [28] and was associated with marked weight loss and intermittent diarrhea. In our study, the small intestinal length could not be evaluated. However, weight loss, when present, was usually mild, as most BCS were higher than 3/9. Furthermore, one case underwent necropsy that did not show any small intestinal shortening. 

Congenital partial colonic agenesis is described in humans. It is a rare condition, more common in males and in which the colon is partially or completely replaced by a dilated pouch. It is, however, often associated with anorectal malformations [29] and usually terminates in a fistulous communication with the genitourinary tract [30].

This study has some limitations. Firstly, a precise reference range of the normal colonic length according to the animal weight is lacking in the literature. It may vary according to the animal’s size and may differ if the measure has been performed on dead or alive individuals. Secondly, because of its retrospective nature, diagnostic criteria were not standardised and the data available for retrospective analysis varied depending on the test performed. It is hard to draw reliable conclusions about the diagnostic test results that might have been more specific of a partial colonic agenesis. Likewise, many of our cases were lost to follow-up and treatment efficiency could not be assessed precisely. Reviewing the abdominal ultrasounds was retrospectively performed on fixed images which might have weakened the potential identification of abnormalities. In addition, at the time of presentation, no specific attention was paid to the research on short colons and subtle indicators might thus have been missed. Thirdly, the stratification between Groups A and B depended on the observation of clinical signs by owners who might have missed mild clinical expression or might have missed changes among pets that had outdoor access. Moreover, as stated before, three cats of Group A were adopted as adults without certainty that clinical signs started young. However, we believe that this article might represent a first step to describe this condition, raise awareness of it and lead to further prospective work to develop a case definition. Finally, discussed complications including stenosis or infections have not been further characterized: full-thickness biopsies would have been needed to thoroughly characterise the stenosis and fluorescence in situ hybridation (FISH) analysis could have been done to investigate invasive bacteria within the colonic mucosa.

## 5. Conclusions

In conclusion, partial colonic agenesis is a congenital abnormality that is occasionally seen in dogs and cats with diarrhea. The age at presentation varies widely, and animals can remain asymptomatic during extended periods of time. Abdominal ultrasonography seems to be a more useful modality to identify this abnormality in cats than in dogs. Colonoscopy is needed to confirm the diagnosis, precisely perform colonic measurement, identify other abnormalities, and perform biopsy sampling required for histopathology. Congenital colonic agenesis might predispose animals to a self-perpetuating inflammation that could ultimately lead to fibrosis and stenosis. After exclusion of the alimentary and infectious causes, early colonoscopy or contrast radiography might be indicated to exclude this disease in young animals with persistent diarrhea. Partial colonic agenesis should be included in the differentials in any dog and cat presenting clinical signs of colonic disease.

## Figures and Tables

**Table 1:** Cases included and epidemiological data (P + M: Praziquantel and Milbemycin oxime; F: fenbendazole; ?: unknown).

Number	Species	Breed	Age (Months)	Sexes	Weight (kg)	Outdoor /Indoor	Deworming	Food Supplied	Vaccination Status
1	Cat	Bengal	16	Female	2.26	Indoor	P + M	Hyperdigestible	Up to date
2	Cat	Savannah	13	Female	2.3	Indoor	F	Hydrolyzed	Up to date
3	Cat	Exotic Shorthair	48	Male	3.2	Indoor	None	Hyperdigestible	Up to date
4	Cat	DSH	160	Male	4.2	Outdoor	P + M	Hyperdigestible	Up to date
5	Cat	Bengal	120	Male	3.6	Indoor	P + M	Urinary diet	Up to date
6	Cat	DSH	11	Male	2.2	?	?	Home-made diet	?
7	Cat	Persian	120	Male	4.3	Indoor	P + M	Hyperdigestible	Up to date
8	Cat	DSH	12	Male	2.7	Indoor	P + M	Hydrolyzed	Up to date
9	Cat	DSH	130	Male	5.1	Outdoor	P + M	?	Up to date
10	Cat	Siberian	12	Male	3.7	?	F	Conventional diet	Up to date
11	Cat	Scottish	6.5	Male	2.3	Indoor	?	Hyperdigestible	Out of date
12	Cat	Chartreux	192	Male	6.3	Outdoor	P + M	Urinary diet	Up to date
13	Cat	Birman	96	Female	2.25	Indoor	P + M	Hydrolyzed	Up to date
14	Cat	Burmese	156	Female	1.9	Indoor	?	Hyperdigestible	Up to date
15	Cat	Abyssinian	81	Female	3.6	Indoor	P + M	Hyperdigestible	Up to date
16	Cat	DSH	103	Female	3.3	Indoor	None	Conventional diet	Out of date
17	Cat	Bengal	18	Female	2.6	Outdoor	F	Hyperdigestible	Up to date
18	Dog	Collie	96	Male	26.5	Outdoor	?	Home-made diet	Up to date
19	Dog	Boxer	4	Female	15	Outdoor	F	Conventional diet	Up to date
20	Dog	French Bulldog	16	Female	9.7	Outdoor	F	Hyperdigestible	Up to date
21	Dog	Whippet	108	Male	10.8	Outdoor	None	Urinary diet	Up to date
22	Dog	French bulldog	60	Female	8.5	Outdoor	P + M	Conventional diet	Up to date
23	Dog	Boxer	52	Female	30.5	Outdoor	P + M	Hyperdigestible	Up to date

**Table 2:** Clinical signs in both groups (y = yes; n = no; N = normal; W = watery; S = soft; M = mixed, D = dysorexia; P = polyphagia; ? = unknown).

Number	Stools (S, W, N, M)	Hematochezia (y/n)	Mucous (y/n)	Urgency to Defecate (y/n)	Dyschezia/ Tenesmus (y/n)	Appetite (N, P, D)	Weight Loss/Failure to Grow (y/n)	Increased Frequency of Defecation (y/n)	Vomiting (y/n)
1	N	y	n	y	y	N	y	y	n
2	W	n	n	y	n	N	y	y	y
3	W	y	y	n	n	N	y	?	y
4	M	y	n	n	n	N	n	n	n
5	M	y	n	n	n	N	n	y	y
6	W	y	n	?	y	N	y	?	y
7	S	y	y	n	n	N	n	y	n
8	M	y	n	y	y	P	y	y	n
9	N	n	n	n	n	D	n	n	y
10	S	n	y	n	y	N	n	y	n
11	W	y	y	y	y	N	n	y	n
12	M	n	n	n	n	D	y	n	n
13	M	n	n	n	n	N	y	y	y
14	W	n	n	n	n	N	y	n	y
15	W	n	n	n	n	N	n	y	n
16	W	n	n	y	n	N	n	y	n
17	W	n	n	y	n	N	y	y	n
18	W	y	n	n	n	N	y	y	n
19	S	y	y	y	y	N	n	?	n
20	S	y	y	y	n	N	n	?	n
21	S	n	n	n	n	D	n	y	n
22	W	n	n	n	n	D	y	n	y
23	S	y	n	n	n	N	n	n	n

**Table 3:** Clinical signs in Group A (y = yes; n = no; N = normal; W = watery; S = soft; M = mixed, D = dysorexia; P = polyphagia; ? = unknown).

Number	Stools (S, W, N, M)	Hematochezia (y/n)	Mucous (y/n)	Urgency to Defecate (y/n)	Dyschezia/ Tenesmus (y/n)	Appetite (N, P, D)	Weight Loss/Failure to Grow (y/n)	Increased Frequency of Defecation (y/n)	Vomiting (y/n)
1	N	y	n	y	y	N	y	y	n
2	W	n	n	y	n	N	y	y	y
3	W	y	y	n	n	N	y	?	y
4	M	y	n	n	n	N	n	n	n
6	W	y	n	?	y	N	y	?	y
7	S	y	y	n	n	N	n	y	n
10	S	n	y	n	y	N	n	y	n
11	W	y	y	y	y	N	n	y	n
13	M	n	n	n	n	N	y	y	y
15	W	n	n	n	n	N	n	y	n
17	W	n	n	y	n	N	y	y	n
19	S	y	y	y	y	N	n	?	n
Percentage/12 cases	N: 8.3%; M: 16.6%; S: 25%; W: 50%	Yes: 58.3%	Yes: 41.6%	Yes: 41.6%	Yes: 41.6%	N: 100%	Yes: 50%	Yes: 66.6%	Yes: 33.3%

**Table 4:** Clinical signs in Group B (y = yes; n = no; N = normal; W = watery; S = soft; M = mixed, D = dysorexia; P = polyphagia; ? = unknown).

Number	Stools (S, W, N, M)	Hematochezia (y/n)	Mucous (y/n)	Urgency to Defecate (y/n)	Dyschezia/ Tenesmus (y/n)	Appetite (N, P, D)	Weight Loss/Failure to Grow (y/n)	Increased Frequency of Defecation (y/n)	Vomiting (y/n)
5	M	y	n	n	n	N	n	y	y
8	M	y	n	y	y	P	y	y	n
9	N	n	n	n	n	D	n	n	y
12	M	n	n	n	n	D	y	n	n
14	W	n	n	n	n	N	y	n	y
16	W	n	n	y	n	N	n	y	n
18	W	y	n	n	n	N	y	y	n
20	S	y	y	y	n	N	n	?	n
21	S	n	n	n	n	D	n	y	n
22	W	n	n	n	n	D	y	n	y
23	S	y	n	n	n	N	n	n	n
Percentage/11 cases	N: 9%; M: 27%; S: 27%; W: 36%	Yes: 45%	Yes: 9%	Yes: 27%	Yes: 9%	N: 54%; D: 36%; P: 9%	Yes: 45.5%	Yes: 45.5%	Yes: 36%

**Table 5:** Severity of colonic wall thickening at diagnosis (abdominal ultrasound).

Severity of Colonic Wall Thickening	Unremarkable	Mild	Moderate	Severe
Group A (/11 cases)	3	3	4	1
Group B (/8 cases)	3	2	2	1

**Table 6:** Endoscopic findings according to the presentation.

	Group A (10 Cases)		Group B (10 Cases)		
Endoscopic Findings	Number	Percentage (/20 Endoscopies)	Number	Percentage (/20 Endoscopies)	*p* Value (Chi-Squared Test)
Ileocolic junction abnormalities	4	20%	4	20%	1
Stenosis	10	50%	2	10%	<0.001
Macroscopic erosion	1	5%	4	20%	<0.001
Nonspecific mild to moderate colopathy	5	25%	3	15%	0.077
Unremarkable	1	5%	1	5%	1

**Table 7:** Histologic findings according to the presentation (LP: lymphoplasmacytic), established according to the WSAVA criteria [18].

	Group A (9 Cases)		Group B (11 Cases)	
Histological Findings	Number	Percentage (/20 Endoscopies)	Number	Percentage (/20 Endoscopies)
Mild-to-moderate LP infiltration	6	30%	10	50%
Marked LP infiltration	2	10%	1	5%
Neutrophilic infiltration	5	25%	4	20%
Fibrosis	4	20%	2	10%
Eosinophilic infiltration	0	0%	2	10%
Erosion/ulcers	3	15%	3	15%

**Table 8:** Diagnosis, treatments and outcomes (AKD: adenocarcinoma, LP: lymphoplasmacytic, FIP: Feline Infectious Peritonitis, IBD: Inflammatory Bowel Disease, PLE: Protein-Losing-Enteropathy, LGITL: Low Grade Intestinal T-cell Lymphoma, D: dog, C: cat, ULF: ultra-low-fat, TMPS: trimethoprim/sulfamethoxazole; PO: per os; SC: sub-cutaneous). Cases are referenced according to Table 1.

Group	Case Number	Diagnosis	Treatment	Outcome
A	1	Subgroup C and stenosis (C)	Colectomy	Died in the post operative period
	2	Subgroup C and stenosis (C)	Bougienage, amoxicillin/clavulanic acid 25 mg/kg q12h PO (Clavaseptin^®^), metronidazole 10 mg/kg q12h PO (Flagyl^®^), prednisolone 0.5 mg/kg/d PO (Microsolone^®^), probiotics (Fortiflora^®^), pancreatic enzymes supplementation 12 500 U/cat q12h PO (Eurobiol^®^), cobalamine 250 µg/cat once a week SC (Vitamine B12 Lavoisier^®^), diosmectite 0.75 g/kg q12h PO (Smecta^®^), hyperdigestible diet	Lost to follow-up
	3	Tritrichomonosis (C)	Ronidazole 30 mg/kg/d PO (pharmaceutical compounding)	Lost to follow-up
	4	Subgroup C and stenosis (C)	Metronidazole 14 mg/kg q12h PO (Metrobactin^®^), prednisolone 0.7 mg/kg/d PO (Microsolone^®^), hyperdigestible diet	Lost to follow-up
	6	Subgroup C and stenosis (C)	Colonic stent and bougienage, intra-rectal lidocaine 2% q12h (Titanoreine^®^), prednisolone 1 mg/kg/d PO (Microsolone^®^), metronidazole 11 mg/kg q12h PO (Eradia^®^), pancreatic enzymes 12,500 U/cat q12h PO (Eurobiol^®^), hyperdigestible diet, diosmectite 0.7 g/kg q12h PO (Smecta^®^)	Marked improvement
	7	Subgroup C with variable degree of LP inflammation (C)	Prendnisolone 0.5 mg/kg/d PO (Dermipred^®^)	Lost to follow up
	10	Short colon with stenosis. Granulomatous infiltrate (FIP suspected) (C)	Colectomy then prednisolone 1.5 mg/kg PO (Dermipred^®^), diosmectite PO if needed 1 g/kg q12h (Smecta^®^), metronidazole 19 mg/kg q12h PO (Metrobactin^®^) and GS 441524 SC	Mild improvement
	11	Subgroup C and stenosis (C)	Metronidazole 8 mg/kg & spiramycin 50,000 U/kg q12h PO (Stomorgyl PA^®^), Lactulose 0.5 mL/kg q12h PO (Laxatract^®^) and a/d food	Marked improvement
	13	Subgroup C and stenosis (C)	Metronidazole 7.5 mg/kg & spiramycin 45,000 U/kg q12h PO (Stomorgyl PA^®^), prednisolone 0.7 mg/kg/d PO (Microsolone^®^), hyperdigestible diet	Lost to follow-up
	15	Subgroup C and stenosis (C)	Prednisolone 0.75 mg/kg/d PO (Dermipred^®^), marbofloxacin 5 mg/kg PO (Marbocyl^®^), psyllium 2.5 g/cat PO (Fiberact^®^), hyperdigestible diet	Recent inclusion
	17	Subgroup C and stenosis (C)	Fiber enriched diet, metronidazole 9.6 mg/kg & spiramycin 58,000 U/kg q12h PO (Stomorgyl PA^®^), prednisolone 1 mg/kg/d PO (Dermipred^®^)	Recent inclusion
	19	Granulomatous colitis (D)	Marbofloxacin 4 mg/kg PO (Marbocyl^®^)	Improvement
B	5	IBD (C)	Prednisolone 0.5 mg/kg PO (Dermipred^®^) and amoxicillin/clavulanic acid 14 mg/kg q12h (Clavaseptin^®^) PO	Lost to follow up
	8	Granulomatous inflammation of unknown origin (C)	Prednisolonone 2 mg/kg/d PO (Dermipred^®^), diosmectite 1 g/kg q8h PO (Smecta^®^), TMPS 15 mg/kg q12h PO (Bactrim^®^)	Died within two weeks of hypovolemic shock
	9	LGITL in ileum (C)	Prednisolonone 2 mg/kg/d PO (Dermipred^®^), chlorambucil 20 mg/m2 every 2 weeks PO (Chloraminophene 2 mg^®^)	Marked improvement
	12	LP colitis (C)	Prednisolone 0.5 mg/kg PO (Microsolone^®^) and hyperdigestible diet	Marked improvement
	14	IBD with cholecystitis (C)	Marbofloxacin 5 mg/kg PO (Marbocyl^®^), amoxicillin/clavulanic acid 18 mg/kg q12h PO (Clavaspetin^®^), prednisolone 0.65 mg/kg/d PO (Microsolone^®^) after cholelithiasis removal.	Marked improvement
	16	IBD with stenotic areas (C)	Prednisolone 0.8 mg/kg PO (Dermipred^®^), marbofloxacin 3.1 mg/kg PO (Marbocyl^®^), psyllium 5g/cat PO (Fiberact ^®^), hyperdigestible diet	Recent inclusion
	18	IBD with stenotic areas (D)	Diosmectite 0.25 g/kg q12h PO (Smecta^®^), homemade eviction diet, probiotics PO (Fortiflora^®^), tylosine 20 mg/kg q12h PO (pharmaceutical compounding), prednisolone 0.5 mg/kg/d PO (Dermipred^®^).	Marked improvement. Colonic ADK diagnosed six months after
	20	Granulomatous colitis (D)	Enrofloxacin 7.4 mg/kg PO (Baytril^®^), fenebendazole 46 mg/kg PO (Panacur^®^), diosmectite 0.5 mg/kg q8h PO (Smecta^®^)	Died of septic shock after aspiration pneumonia
	21	Granulomatous colitis (D)	Marbofloxacin 4 mg/kg PO (Marbocyl^®^)	Marked improvement
	22	PLE (D)	Prednisolone 2 mg/kg/d PO (Dermipred^®^), cobalamine 55 µg/kg every other day PO (Cobalaplex^®^), ciclosporine 5 mg/kg/d PO (Atopica^®^), clopidogrel PO (Plavix^®^), metronidazole PO (Metrobactin^®^), ULF diet	Improvement with normal stools
	23	Granulomatous colitis (D)	Enrofloxacin 5 mg/kg PO (Baytril^®^), hyperdigestible diet, diosmectite 0.3 g/kg q8h PO (Smecta^®^)	Marked improvement

## Data Availability

The data presented in this study are available on request from the corresponding author. The data are not publicly available due to privacy but are reachable on the Veterinary Teaching Hospital Software.
